# Supplementary material for: Advancing PEDV Vaccination: Comparison between Inactivated and Flagellin N-Terminus-Adjuvanted Subunit Vaccines
Source: Vaccines (Basel). 2024 Jan 29;12(2):139. doi: 10.3390/vaccines12020139 (PMC10892538; doi:10.3390/vaccines12020139)
Supplement: Supplementary file 1 [file vaccines-12-00139-s001.zip › vaccines-2779037-supplementary.pdf]

Supplementary Data

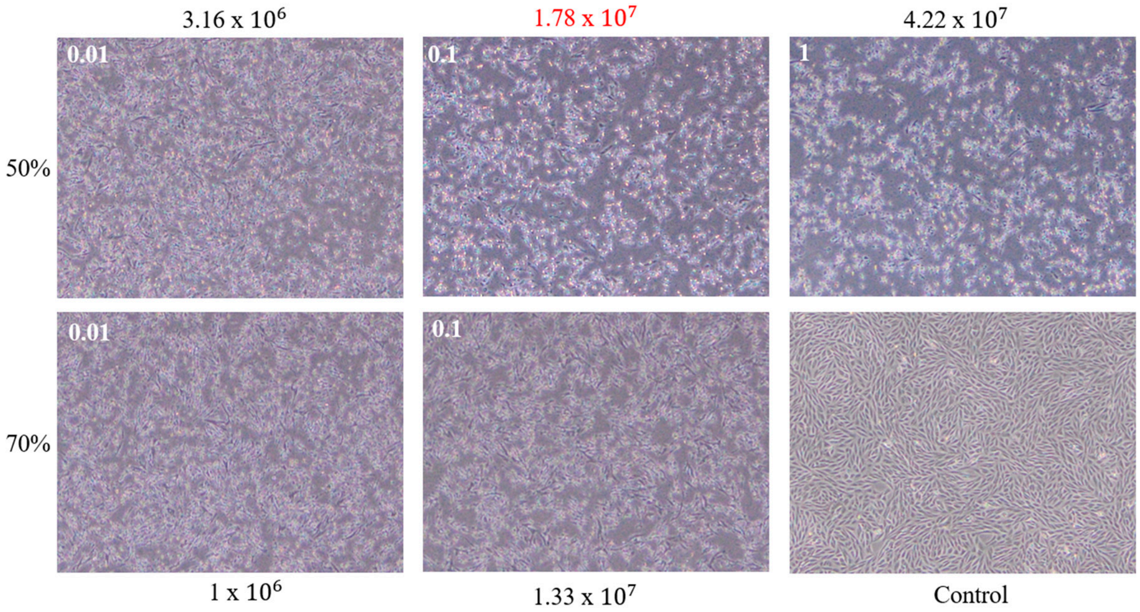

Figure S1: PEDV infection in different MOI concentrations

To determine the optimal MOI concentration and the cell confluence for virus production. PEDV was inoculated at different MOI (0.01, 0.1, 1) and different confluence (50 and 70%). Then cell viability and the morphological changes in PEDV infected cells were observed by microscopy. PEDV infected at 65 hours post-infection and the result of TCID_{50} was showed.

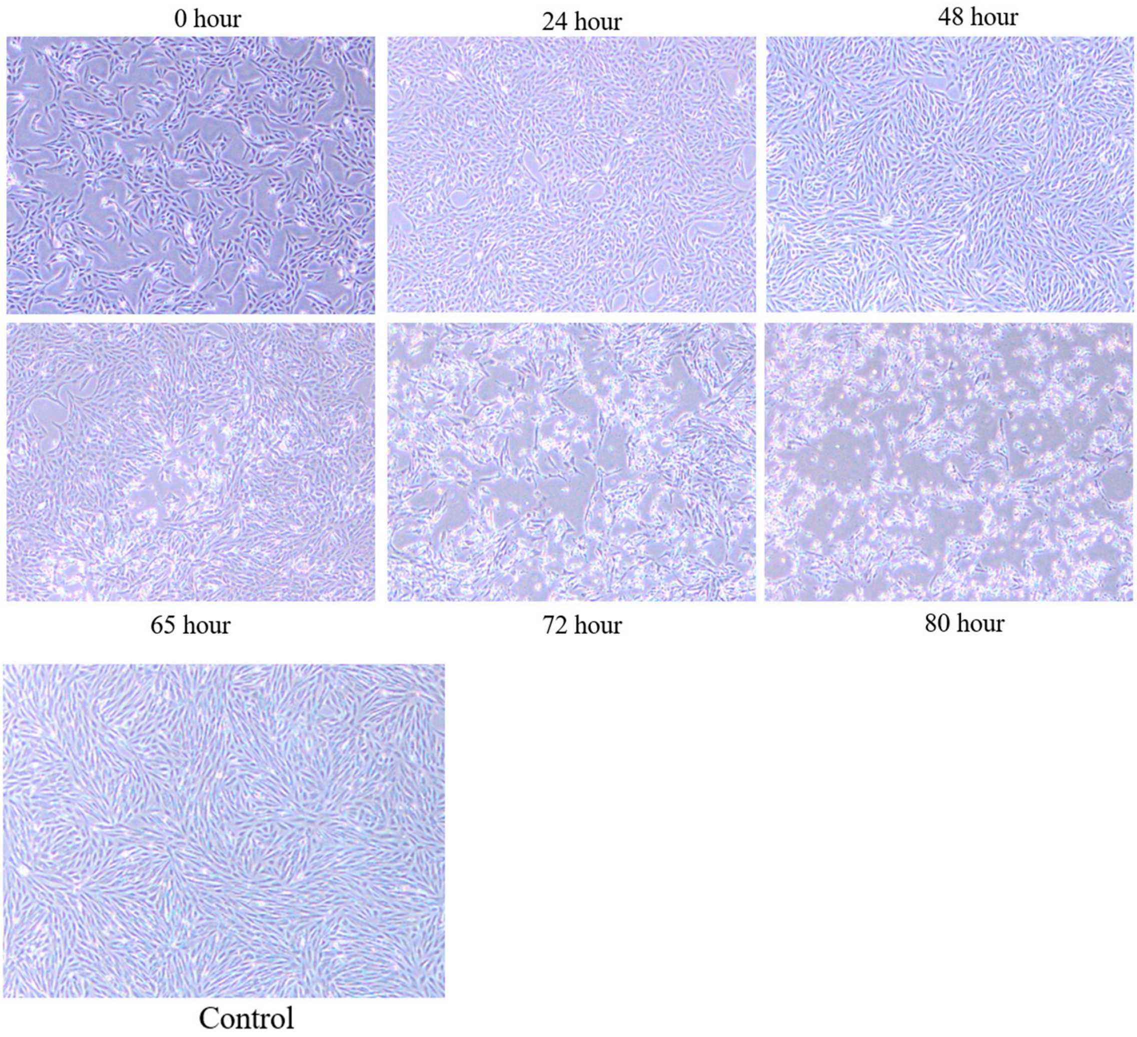

Figure S2: PEDV infection reduced viability in Vero cells.

To determine the time of collection after infection, cultured Vero cell was infected with PEDV. Then cell viability and the morphological changes in PEDV infected cells at 0.1 MOI were observed at different time points: 0, 24, 48, 65, 72 and 80 hours after infection by microscopy.

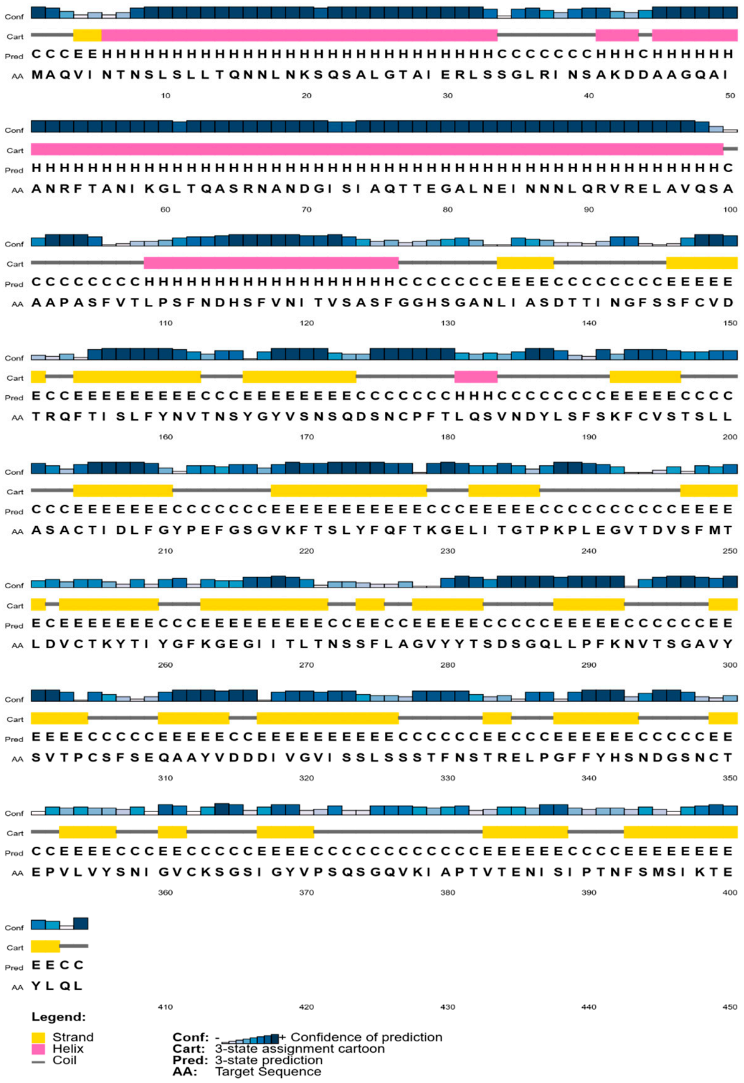

Figure S3: Psipred predicted secondary structure of vaccine.

Rank	Sequence	Start position	Antigenicity
1	GELITGTPKPLEGVTD	230	0.96
2	SFSEQAAYVDDDIVGV	306	0.92
3	TIDLFGYPEFGSGVKF	205	0.90
4	DGISIAQTTEGALNEI	70	0.88
4	NVTNSYGYVSNSQDSN	161	0.88
5	LGTAIERLSSGLRINS	25	0.87
6	LQSVNDYLSFSKFCVS	181	0.86
7	TVSASFGGHSGANLIA	121	0.85
8	GQVKIAPTVTENISIP	375	0.84
9	VGVISSLSSSTFNSTR	319	0.83
9	SGVKFTSLYFQFTKGE	216	0.83
10	TQASRNANDGISIAQT	62	0.82
11	QTTEGALNEINNNLQR	76	0.81
11	SFLAGVYYTSDSGQLL	274	0.81
11	LEGVTDVSFMTLDVCT	240	0.81
12	GLRINSAKDDAAGQAI	35	0.80
13	SGSIGYVPSQSGQVKI	364	0.78
13	AVYSVTPCSFSEQAAY	298	0.78
13	NKSQSALGTAIERLSS	19	0.78
14	DVCTKYTIYGFKGEGI	252	0.77
14	TSLLASACTIDLFGYP	197	0.77
15	DDAAGQAIANRFTANI	43	0.75
15	DTRQFTISLFYNVTNS	150	0.75
15	ANLIASDTTINGFSSF	132	0.75
15	ASFVTLPSFNDHSFVN	104	0.75

Rank	Sequence	Start position	Antigenicity
16	AVQSAAAPASFVTLPS	96	0.74
16	PGFFYHSNDGSNCTEP	337	0.74
16	YTSDSGQLLPFKNVTS	281	0.74
16	AQVINTNSLSLLTQNN	2	0.74
16	SNSQDSNCPFTLQSVN	170	0.74
17	NCTEPVLVYSNIGVCK	348	0.71
17	KGEGIITLTNSSFLAG	263	0.71
17	SKFCVSTSLLASACTI	191	0.71
18	TENISIPTNFSMSIKT	384	0.69
19	DTTINGFSSFCVDTRQ	138	0.68
20	LNEINNNLQRVRELAV	82	0.65
21	QLLPFKNVTSGAVYSV	287	0.61

Table S1: Linear cell epitopes of vaccine construct, predicted by ABCPred server.

Cluster	Residues	Size	Z-Score
1	A:V377, A:K378, A:I379, A:A380, A:P381, A:T382, A:V383, A:T384, A:E385, A:N386, A:I387, A:S388, A:I389, A:P390, A:T391, A:N392, A:F393, A:S394, A:M395, A:S396, A:I397, A:K398, A:T399, A:E400, A:Y401, A:L402, A:Q403, A:L404	28	0.954
2	A:Y260, A:L270, A:T271, A:N272, A:S273, A:S274, A:F275, A:L276, A:A277, A:G278, A:V279, A:Y280, A:Y281, A:K292, A:N293, A:V294, A:T295, A:S296, A:G297, A:A298, A:V299, A:Y300	22	0.756
3	A:T108, A:L109, A:P110, A:S111, A:F112, A:N113, A:D114, A:H115, A:S116, A:T121, A:V122, A:S123, A:A124, A:S125, A:F126, A:G127, A:G128, A:H129, A:S130, A:G131, A:A132, A:N133, A:L134, A:I135, A:A136, A:S137, A:D138, A:T139, A:T140, A:I141, A:N142, A:G143, A:F144, A:S145, A:S146, A:F147, A:C148, A:V149, A:D150, A:T151, A:R152, A:N161, A:V162, A:T163, A:N164, A:S165, A:Y166, A:G167, A:Y168, A:V169, A:S170, A:N171, A:F190, A:S191, A:K192, A:C194, A:S196, A:T197, A:S198, A:L199, A:L200, A:A201, A:S202, A:A203, A:F209, A:G210, A:Y211, A:P212, A:E213, A:F214, A:G215, A:S216, A:G217, A:V218, A:K219, A:F220, A:T221, A:S222, A:Y224, A:Q226, A:T228, A:K229, A:G230, A:E231, A:L232, A:K238, A:P239, A:L240, A:E241, A:G242, A:V243, A:T244, A:D245, A:V246, A:S247, A:F248, A:M249, A:T250, A:L251	99	0.682
4	A:K20, A:Q22, A:S23, A:A24, A:L25, A:G26, A:T27, A:A28, A:I29, A:E30, A:R31, A:L32, A:S33, A:S34, A:G35, A:L36, A:R37, A:I38, A:N39, A:S40, A:A41, A:K42, A:F54	23	0.652
5	A:R53, A:T55, A:A56, A:N57, A:I58, A:K59, A:Y313, A:V314, A:D316, A:D317, A:I318, A:V319, A:G320, A:F330, A:N331, A:S332, A:T333, A:R334, A:S343, A:N344, A:D345, A:G346, A:S347, A:N348, A:C349, A:T350, A:E351, A:P352, A:V353, A:S357, A:N358, A:C362, A:K363, A:S364, A:G365, A:S366, A:I367, A:G368, A:Y369, A:V370, A:P371, A:S372, A:Q373, A:S374, A:G375, A:Q376	46	0.611
6	A:G60, A:L61, A:T62, A:Q63	4	0.505

Table S2: Conformational BCEs, identified by Ellipro server.

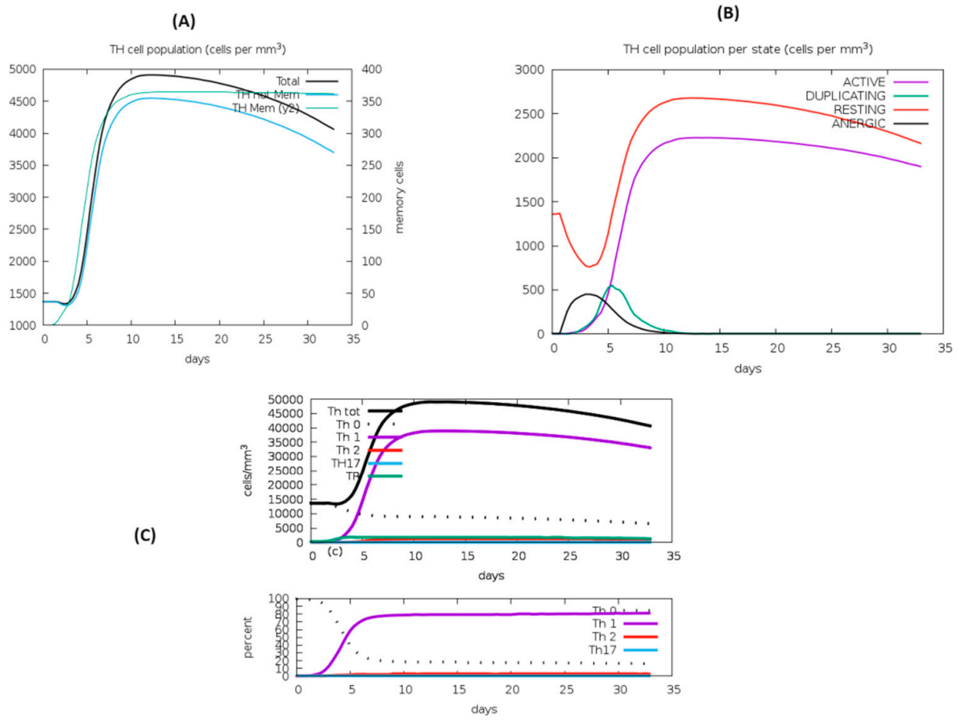

Figure S4: In-silico analysis Immune response to the vaccine.

The immune response details are depicted in the figure, with part (A) illustrating the overall T helper cell population. Part (B) differentiates this population based on cellular state, categorizing cells as active, duplicating, or resting. Part (C) provides a breakdown of the T helper cell types and their respective responses, presented as percentages.

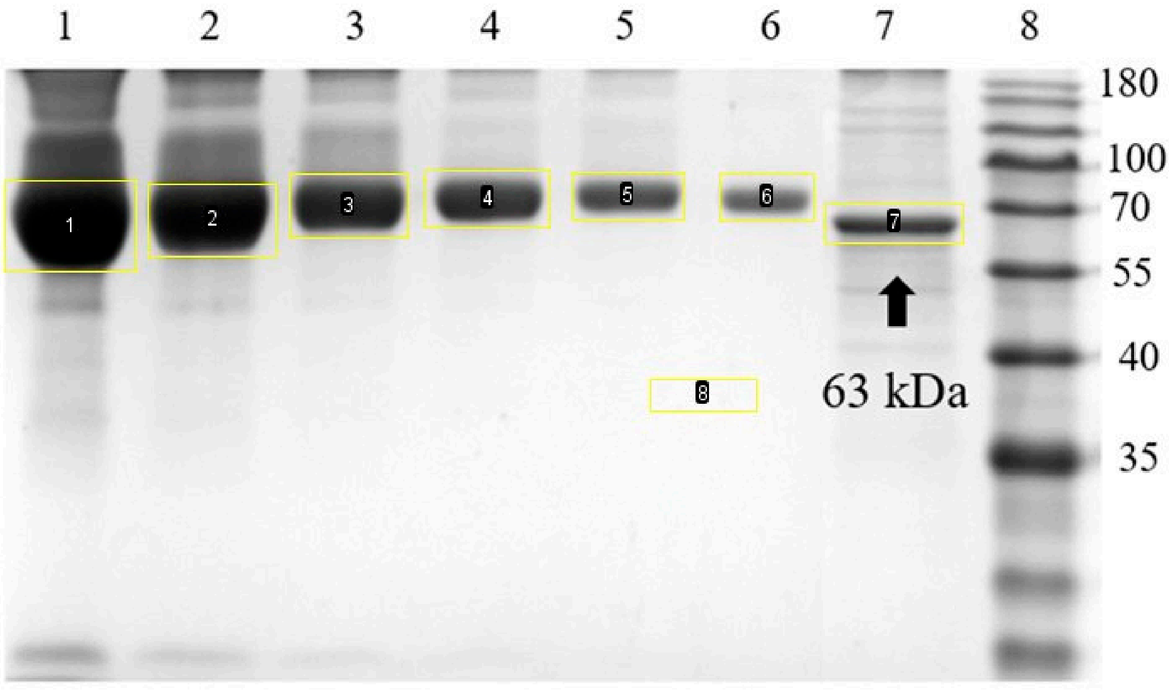

	Area	Mean	integrated density	Raw integrated density
1	4212	60.348	254184	254184
2	3268	63.533	207625	207625
3	2698	98.185	264902	264902
4	2516	128.413	323087	323087
5	1876	147.257	276255	276255
6	1568	174.002	272835	272835
7	1886	151.782	286261	286261
8	1197	249.936	299173	299173

Figure S5 and Table S3: Western Blot analysis displaying the FliC$_{99}$-*m*COE protein and various concentrations of bovine serum albumin (BSA), accompanied by a table showing the density ratios of the respective bands.

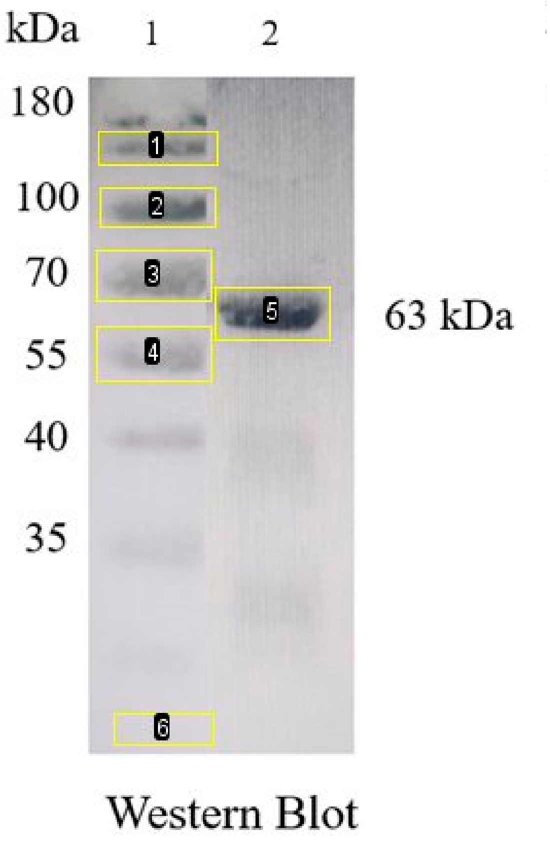

	Area	Mean	integrated density	Raw integrated density
1	1037	154.777	160504	160504
2	1180	152.071	179444	179444
3	1534	172.980	265352	265352
4	1652	188.979	312193	312193
5	1593	131.562	209578.000	209578.000
6	816	212.273	173215.000	173215.000

Figure S6 and Table S4: Western blot analysis depicting FliC$_{99}$-*m*COE, complemented by a table detailing the density ratios of the observed bands.

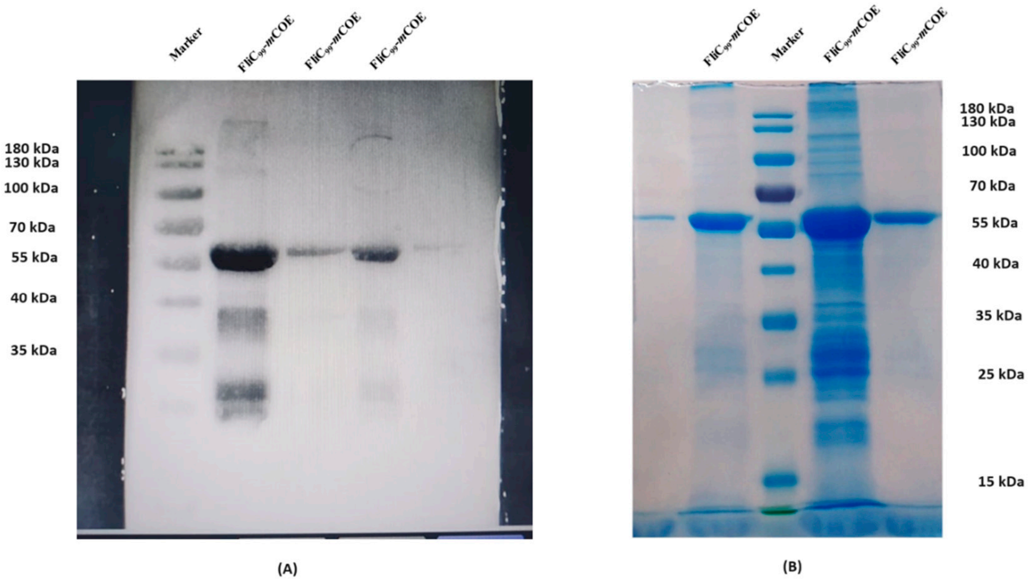

Figure S7: Uncropped western blot (A) and SDS-PAGE (B) photos of the recombinant protein (FliC$_{99}$-*m*COE).
